# Electronic bonding analyses and mechanical strengths of incompressible tetragonal transition metal dinitrides TMN_2_ (TM = Ti, Zr, and Hf)

**DOI:** 10.1038/srep36911

**Published:** 2016-11-10

**Authors:** Meiguang Zhang, Ke Cheng, Haiyan Yan, Qun Wei, Baobing Zheng

**Affiliations:** 1 College of Physics and Optoelectronic Technology, Nonlinear Research Institute, Baoji University of Arts and Sciences, Baoji 721016, China; 2 College of Optoelectronic Technology, Chengdu University of Information Technology, Chengdu 610225, China; 3 College of Chemistry and Chemical Engineering, Baoji University of Arts and Sciences, Baoji 721013, China; 4 School of Physics and Optoelectronic Engineering, Xidian University, Xi’an 710071, China

## Abstract

Motivated by recent successful synthesis of transition metal dinitride TiN_2_, the electronic structure and mechanical properties of the discovered TiN_2_ and other two family members (ZrN_2_ and HfN_2_) have been thus fully investigated by using first-principles calculations to explore the possibilities and provide guidance for future experimental efforts. The incompressible nature of these tetragonal TMN_2_ (TM = Ti, Zr, and Hf) compounds has been demonstrated by the calculated elastic moduli, originating from the strong N-N covalent bonds that connect the TMN_8_ units. However, as compared with traditional *fcc* transition metal mononitride (TMN), the TMN_2_ possess a larger elastic anisotropy may impose certain limitations on possible applications. Further mechanical strength calculations show that tetragonal TMN_2_ exhibits a strong resistance against (100)[010] shear deformation prevents the indenter from making a deep imprint, whereas the peak stress values (below 12 GPa) of TMN_2_ along 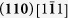
shear directions are much lower than those of TMN, showing their lower shear resistances than these known hard wear-resistant materials. The shear deformation of TMN_2_ at the atomic level during shear deformation can be attributed to the collapse of TMN_8_ units with breaking of TM-N bonds through the bonding evolution and electronic localization analyses.

Transition metal nitrides (TM_x_N_y_), synthesized under high-pressure and high-temperature conditions, represent a prominent class of materials exhibiting extreme usefulness in a wide variety of industrial applications^1^,^2^. When it comes to their superior mechanical properties such as high hardness and elastic moduli, most of the early transition metal mononitrides (TMN), and in particular TiN and CrN are well known hard materials and are widely used in various industrial applications, such as cutting tools or wear-resistant coatings^3^,^4^. Taking advantage of high-pressure techniques, two family members of hard nitrides (Zr_3_N_4_ and Hf_3_N_4_) of the group IVB with TM_3_N_4_ stoichiometry were successfully synthesized^5^, opening a promising way to obtain other nitrides with N:TM > 1 under high nitrogen pressure. Compared to early transition metals, the noble metals (TM = Ru, Rh, Pd, Os, Ir, and Pt) were previously known to hardly form nitrides with high nitrogen content. Until 2004, a novel platinum nitride with ultra-high incompressibility was obtained under extreme conditions (50 GPa and 2000 K) by Gregoryanz *et al*.^6^ and was finally determined to crystallize in the pyrite structure with a stoichiometry of 1:2^7^,^8^. Thereafter, there have been considerable researches to search for other transition metal dinitrides, and so far as we know, the OsN_2_^8^,^9^, IrN_2_^7^,^9^,^10^, PdN_2_^10^, RhN_2_^11^, and recently RuN_2_^12^ have been experimentally obtained in a direct chemical reaction between platinum group elements and molecular fluid nitrogen at high pressures and temperatures. The follow-up studies of their structures and mechanical properties have stimulated significant in their potential applications. These works have been motivated by the design of intrinsic (super)hard materials proposed by Kaner *et al*.^13^,^14^ that the introduction of light and covalent-bond-forming elements, such as B, C, N, and O into the transition metal (TM) lattices with highly valence-electron density is expected to enhance the shear strength against plastic deformations.

More recently, a new transition metal dinitride, TiN_2_^15^, was successfully synthesized at 73(3) GPa and 2400(40) K by choice of TiN and dense N_2_ as starting materials. The experiment revealed that this new dinitride adopts a tetragonal CuAl_2_-type structure at high pressure, which is in agreement with previous theoretical prediction performed by Yu *et al*.^16^. On decompression, the experiment found that this phase is recoverable to ambient conditions and possesses a high bulk modulus of 385(7) GPa comparable to those of PtN_2_ (372 GPa)^6^ and ReB_2_ (360 GPa)^17^, much larger than that of TiN (288 GPa)^18^. Therefore, this new tetragonal TiN_2_, the first synthesized high-nitride phase in early transition metal nitrides, is expected to be a candidate as a potential superhard solid for wear- and scratch-resistant materials. However, this concept for the search of novel superhard materials failed in materials such as PtN_2_^19^, and ReB_2_^20^, and others^21^,^22^,^23^, because plastic deformation occurs in shear at large strain at the atomic level, where electronic instabilities may occur upon bond breaking in the practical measurement of hardness. Meanwhile, the macroscopic behavior of a solid is strongly related to its elastic anisotropic properties, which can reveal, in some materials, an anisotropy degree decidedly non-negligible and in some cases so extreme to suggest the proximity of material instability. Accordingly, here, we have extended the mechanical behaviors of TiN_2_ and presented in detail the variations of the elastic moduli along the arbitrary directions. Moreover, the stress-strain relations and the underlying atomistic bond breaking processes under the applied strains were also systematically investigated to provide a deeper insight into mechanical properties and hardness of the newly discovered TiN_2_. We have also applied this novel tetragonal structure to other two family members ZrN_2_ and HfN_2_ to explore the possibilities and provide guidance for future experimental efforts. We hope that the present findings will encourage further theoretical and experimental works on this class of material.

## Results and Discussion

**Figure 1:**
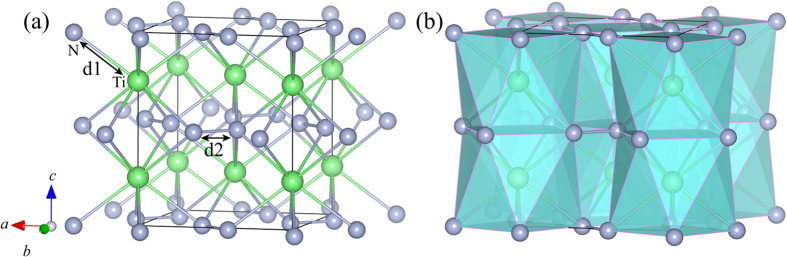
(**a**) Crystal structure of tetragonal TiN_2_ and (**b**) its polyhedral view. The large and small spheres represent Ti and N atoms, respectively.

**Figure 2:**
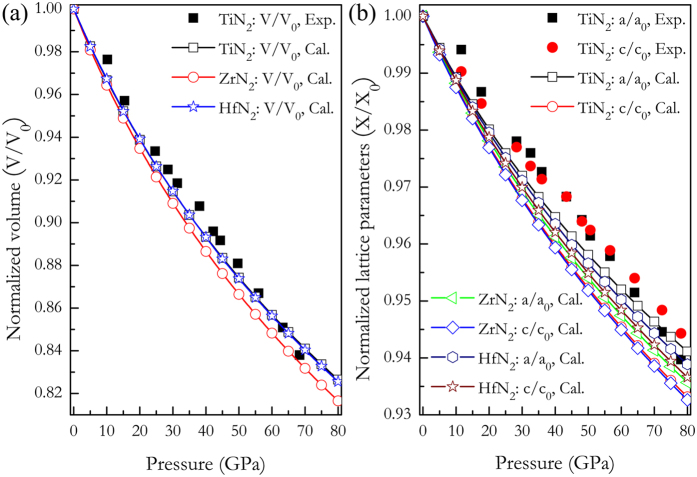
(**a**) The calculated normalized volumes and (**b**) lattice parameters as a function of pressure for tetragonal TMN_2_ compounds.

The experiment has demonstrated that TiN_2_ crystallizes in the tetragonal CuAl_2_-type structure with Ti and N atoms sitting at 4*a* and 8 *h* sites in a unit cell, as shown in Fig. 1(a). Polyhedral view of this tetragonal structure (Fig. 1(b)) reveals that TiN_2_ consists of the TiN_8_ face-sharing tetragonal antiprisms connected by N-N bonds and stacked along the *c*-axis, in contrast to the TMN_6_ octahedrons in the previous synthesized noble metals pernitrides^7^,^8^,^9^,^10^,^11^,^12^. Through the full relaxations of both lattice constants and internal atomic coordination, the obtained equilibrium structure parameters for three TMN_2_ compounds are listed in Table 1, among which the calculated results for TiN_2_ compare well with the available experimental data^15^. For ZrN_2_ and HfN_2_, however, there are no available experimental data for comparison and the present results could provide useful information for further experimental or theoretical investigations. According to the recent experiment by Bhadram *et al*.^15^, the pressure dependences of unit cell volume and lattice constants of TiN_2_ were calculated and plotted in Fig. 2, along with the experimental data^15^ and theoretical results of ZrN_2_ and HfN_2_. First, one can see that the calculated results for TiN_2_ are in agreement with the experimental data under pressure, and the incompressibility of TiN_2_ (Fig. 2(a)) is almost identical to that of HfN_2_, but larger than that of ZrN_2_. Furthermore, from Fig. 2(b), it can be seen that the incompressibility along the *a*-axis is larger than that along the *c* axis for each TMN_2_ compound, indicating their clear elastic anisotropy. Second, the *E*-*V* data under pressures deduced from the Fig. 2(a) for each TMN_2_ are fitted to the third order Birch-Murnaghan equation of state (EOS)^24^. The obtained the bulk modulus (*B*_0_) and its pressure derivative (*B*_0_′) for TiN_2_ are 276 GPa and 4.362 (see Table 1), which are lower than those of experimental data (385 GPa and 1.45), but consistent with the theoretical values (293 GPa and 3.7) predicted by Bhadram *et al*. using the same approach^15^. The low value of *B*_0_′ related to *B*_0_ in this discrepancy has been elucidated in this experimental work. Third, the fitted *B*_0_ values of TiN_2_ and HfN_2_ are nearly equivalent but larger than that of ZrN_2_, which is in accord with the calculated compressibility of volume plotted in Fig. 2(a). Overall, the accuracy of the present calculations for TiN_2_ is made quite satisfactory with the experimental data in Table 1 and Fig. 2, which supplies the safeguard for the following studies.

**Figure 3:**
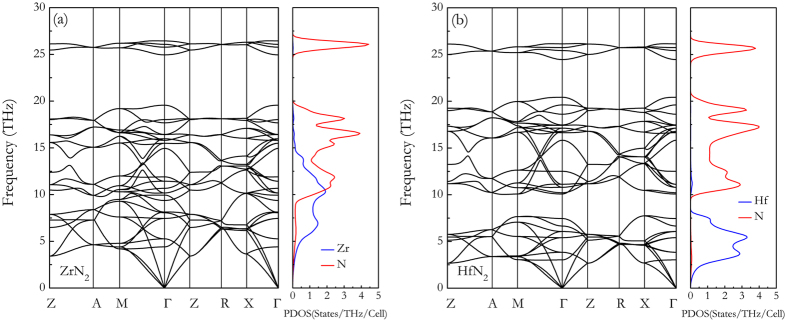
Phonon dispersion curves of TMN_2_ at ambient pressure: (**a**) ZrN_2_ and (**b**) HfN_2_.

According to synthetic conditions of TiN_2_ proposed by Bhadram *et al*.^15^, the thermodynamic feasibility of ZrN_2_ and HfN_2_ is evaluated through the formation enthalpy (energy) calculations. The formation enthalpy Δ*H*_*f*_ of each TMN_2_ with respect to the TMN and nitrogen at ambient conditions based on the reaction route: 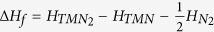
was quantified, where the *fcc* TMN phase and *α*-N_2_ phase are chosen as the reference phases. As listed in Table 1, the calculated formation enthalpies of three TMN_2_ dinitrides are all positive values, indicating that they are all metastable at ambient conditions. It is to be noted that the calculated formation enthalpies of ZrN_2_ (0.372 eV/atom) and HfN_2_ (0.328 eV/atom) are all close to that of TiN_2_ (0.398 eV/atom), which has been synthesized at 73(3) GPa and 2400(40) K by choice of TiN and dense N_2_ as starting materials. Thus, the syntheses of the ZrN_2_ and HfN_2_ could be expected at similar high pressure and temperatures conditions. The experiment has suggested that TiN_2_ can be quenchable to ambient conditions, and the dynamical stabilities of ZrN_2_ and HfN_2_ at 0 GPa have been thus carefully checked by the full phonon dispersions calculations using the 2 × 2 × 2 supercell method. Figure 3(a,b) show the phonon dispersion curves which confirm the dynamic stability of ZrN_2_ and HfN_2_ as there are no imaginary modes in the whole Brillouin zone. The lower frequencies of the phonon density of states are dominated by lattice dynamics of heavy TM atoms and higher frequencies by light N atoms.

**Figure 4:**
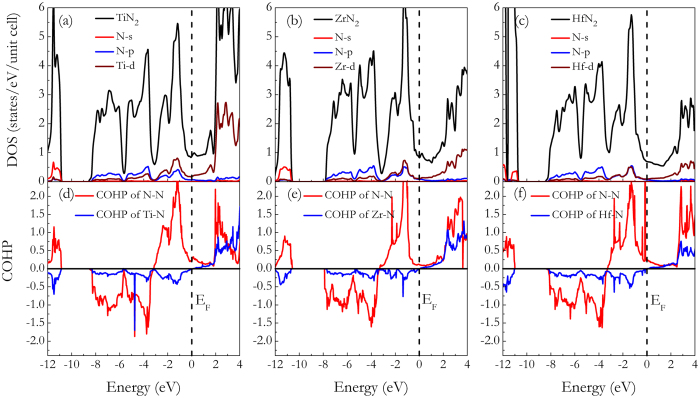
Total and projected DOS of TMN_2_: (**a**) TiN_2_, (**b**) ZrN_2_, and (c) HfN_2_. Projected COHP curves of various bonds in TMN_2_: (**d**) TiN_2_, (**e**) ZrN_2_, and (**f**) HfN_2_. The Fermi level (E_F_) is indicated by vertical dashed lines.

The total and projected density of states (DOS) of each TMN_2_ at ambient pressure was plotted to further elaborate the electronic bonding feature, as shown in Fig. 4(a–c), respectively. All TMN_2_ compounds show metallic bonding because of finite value of DOS at the Fermi level (E_F_), which originates mostly from the TM-*d* orbitals and the N-*p* orbitals. The major orbital occupancy in the energy range of −8–0 eV stems from the strong hybridized states of TM-*d* and N-*p* orbitals, as the usual cases in the most TM_x_N_y_ compounds. The typical feature of the total DOS is the presence of a “pseudogap” (a sharp valley around the E_F_), which is supposed the borderline between the bonding and antibonding states^25^,^26^,^27^. For TiN_2_, it is noteworthy that the bonding states are completely filled with the Fermi energy located exactly at the “pseudogap”. For ZrN_2_ and HfN_2_ (see Fig. 4(b,c)), it is found that the E_F_ shifts toward the higher energy and lies left at the pseudogap with a relative more electronic density of states [N(E_F_)]. It is known that for the most stable structure there is enough room to accommodate all its valence electrons into bonding states so as to bring the E_F_ to a valley position separating bonding and antibonding states (pseudogap) favorable for structural stability. Therefore, the TiN_2_ is energetically more favorable compared to the ZrN_2_ and HfN_2_ in the tetragonal phase. Figure 4(d–f) offers the calculated crystal overlap Hamilton population (COHP)^28^ for the TM-N and the N-N bonding inside TiN_2_, ZrN_2_, and HfN_2_, respectively. For the TM-N combinations in all plots, there are only bonding states in the entire occupied regions, and antibonding states show up in the unoccupied crystal orbitals, well above the E_F_. For the N-N combinations in TMN_2_, the antibonding 1*π*_g_^*^ states (starting near −3.5 eV) are almost completely occupied at the top of the conduction band, and a portion of the metallic nature can be ascribed to these states being occupied at the E_F_. For TiN_2_, this point has been addressed in a recent work by Yu *et al*.^16^. As demonstrated in previous work^29^, for the case of PtN_2_, charge transferred from Pt to N (1.05 *e*) results in the full filling of antibonding 1*π*_g_^*^ states of N_2_^4−^ and leads to the elongation of N-N bonds. In a similar way, this mechanism is also applicable to the case of tetragonal TiN_2_ although antibonding states are not completely filled and there are differences in electronic and structural configurations, as suggested by Bhadram *et al*.^15^ and Yu *et al*.^16^. Consequently, a charge balance of N_2_^4−^ in these TMN_2_ is a good working hypothesis, and this leaves the TM atoms in TMN_2_ in a *d*^0^ configuration. In order to compare the “ionicity” of the three dinitrides, we also analyzed the charge density topology through the Bader charge analyses^30^. The calculated charges of the three nitrides show decreasing trends from Hf ^2.13^N_2_^−2.13^ to Zr^1.96^ N_2_^−1.96^ and Ti^1.75^N_2_^−1.75^, indicating the relatively lower polarity of Ti-N bond. Meanwhile, it has been demonstrated^31^ that the shortening of the N-N bond is ascribed to the decrease in charge transfer from TM to N (*q*_trans_) when one monitors the pernitrides from early to late TM elements. It can be seen that as the TM element moves from Hf through Zr to Ti, as *q*_trans_ from 2.13 *e* through 1.96 *e* to 1.75 *e*, and as *d*_N-N_ from 1.461 Å through 1.434 Å to 1.385 Å.

**Figure 5:**
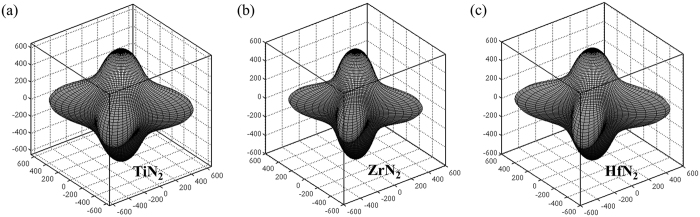
Three-dimensional surface representations of the Young’s modulus *E* for TMN_2_: (**a**) TiN_2_, (**b**) ZrN_2_, and (**c**) HfN_2_.

**Figure 6:**
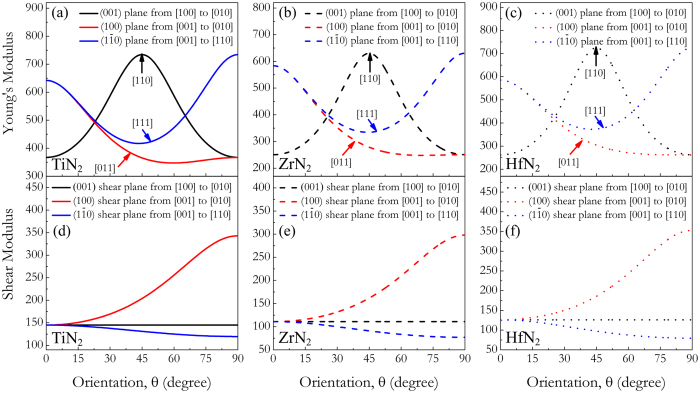
Orientation dependences of the Young’s modulus *E* for TMN_2_: (**a**) TiN_2_, (**b**) ZrN_2_, (**c**) and HfN_2_. Orientation dependence of the Shear modulus *G* for TMN_2_: (**d**) TiN_2_, (**e**) ZrN_2_, and (**f**) HfN_2_.

For potential engineering applications, the elastic stabilities, incompressibility, and rigidity of three TMN_2_ dinitrides are determined from the calculated elastic constants by applying a set of given strains with a finite variation between −0.01 and +0.01. Table 2 summarizes the calculated single-crystal elastic constants *C*_*ij*_ and derived Hill elastic moduli as well as Poisson’s ratios of TMN_2_ dinitrides and compares them with those of typical hard substances TMN (TM = Ti, Zr, and Hf)^32^,^33^,^34^,^35^. The calculated six independent elastic constants of TiN_2_ agree well with recent theoretical results^16^, and the derived bulk moduli of three TMN_2_ dinitrides also accord well with those directly obtained from the fitting of the third-order Birch-Murnaghan EOS (see Table 1), demonstrating the reliability of the present calculations. The mechanical stabilities of three dinitrides satisfy the Born-Huang criterion^36^ for a tetragonal crystal [*C*_11_ > 0, *C*_33_ > 0, *C*_44_ > 0, *C*_66_ > 0, (*C*_11_ − *C*_12_) > 0, (*C*_11_ + *C*_33_ − 2*C*_13_) > 0, and 2(*C*_11_ + *C*_12_) + *C*_33_ + 4*C*_13_ > 0], indicating their mechanically stable at ambient conditions. From Table 2, the high-incompressible nature of TMN_2_ is disclosed by the calculated bulk modulus (TiN_2_: 276 GPa, ZrN_2_: 250 GPa, HfN_2_: 275 GPa), originating from the covalent TMN_8_ polyhedrons connected by the strong N-N covalent bonds in systems. Meanwhile, these values are comparable with the corresponding theoretical calculations and experimental data (in brackets) of typical hard transition metal mononitrides TMN, TiN: 278 GPa (288 GPa), ZrN: 250 GPa (215 GPa), HfN: 273 GPa (306 GPa). The critical values of the ratio of shear modulus *G* to bulk modulus *B* of about 0.57 separates brittle (*G*/*B* > 0.57) and ductile (*G*/*B* < 0.57) materials. For three TMN_2_ dinitrides, their *G*/*B* values (TiN_2_: 0.707, ZrN_2_: 0.608, HfN_2_: 0.626) are all larger than 0.57, implying that they are intrinsically brittle. The theoretical Vickers hardness *H*_*v*_ of each TMN_2_ was estimated by using the Chen’s empirical model^37^, *H*_*v*_ = 2(*k*^2^*G*)^0.585^ − 3. The calculated hardness value for TiN_2_, ZrN_2_, and HfN_2_ is 26.1 GPa, 18.1 GPa, and 23.2 GPa, respectively, making them potentially interesting for applications as hard coating materials. By using the Bader atoms-in-molecules (AIM) method, the strong covalent nature of the N-N and TM-N bonds in TMN_2_ were quantitatively revealed by the evidences of local charge densities 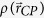 at their bond critical points (BCPs) with negative Laplacian values. The obtained 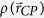 who can measure the bond strength related to the mechanical behaviors located at N_2_ dumbbells and TM-N bonds decrease in the sequence of TiN_2_: (2.324 *e*/Å^3^) > ZrN_2_ (2.047 *e*/Å^3^) ≈ HfN_2_: (2.045 *e*/Å^3^) and TiN_2_: (0.457 *e*/Å^3^) > HfN_2_: (0.444 *e*/Å^3^) > ZrN_2_ (0.428 *e*/Å^3^), respectively. Therefore, compared to TiN_2_ and HfN_2_, the ZrN_2_ exhibits the lowest moduli and hardness. Next we investigate the mechanical anisotropy of tetragonal TMN_2_ by calculating the orientation dependences of the Young’s modulus *E* and shear modulus *G* which can be determined from the elastic compliance constants *s*_*ij*_^38^. The computational details of elastic moduli-crystal orientation dependences conducted here are presented in the Supporting information section. Figure 5 illustrates the three-dimensional surface representation showing the variation of Young’s modulus with direction for each dinitride. Clearly, all three TMN_2_ dinitrides exhibit a well-pronounced elastic anisotropy due to their three-dimensional pictures show a large deviation from the spherical shape, which qualifies an isotropic medium. From Fig. 5(a–c), the calculated *E*_*max*_/*E*_*min*_ ratio of the Young’s moduli for TiN_2_, ZrN_2_, and HfN_2_ is 2.115, 2.543, 2.766, respectively. The *E*_*max*_/*E*_*min*_ ratios for TiN_2_ and ZrN_2_ are much larger than those of *fcc* TiN (1.148) and ZrN (1.539) proposed by Brik *et al*.^39^, suggesting that the TMN_2_ with a larger elastic anisotropy may impose certain limitations on their possible applications. More specifically, the directional Young’s moduli along tensile axes within (001), (100), and 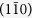 specific planes are plotted in Fig. 6(a–c). For example, the variation of Young’s modulus in the (001) crystal plane for the quadrant of directions [*uvw*] between [100] (*θ* = 0°) and [010] (*θ* = 90°), the TiN_2_/ZrN_2_/HfN_2_ exhibits a maximum of *E*_[110]_ = 734/631/725 GPa and a minimum of *E*_[100]_ = *E*_[010]_ = 367/250/262 GPa, respectively. From Fig. 6(a–c), the ordering of Young’s modulus as a function of direction for three TMN_2_ dinitrides is *E*_[110]_ > *E*_[001]_ > *E*_[111]_ > *E*_[011]_ > *E*_[100]_. Similarly, the orientation dependences of the shear modulus *G* were also conducted for shear on (001), (100), and
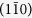
planes. From Fig. 6(d), the shear modulus of the TiN_2_ is independent of the shear stress from [100] to [010] directions within (001) basal plane, and the TiN_2_ possesses its minimum value for shear on 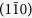
[110] 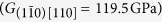 and its maximum value for shear on (100)[010] (*G*_(100)[010]_ = 343 GPa). The similar cases can be also found for ZrN_2_ and HfN_2_ in Fig. 6(e,f).

**Figure 7:**
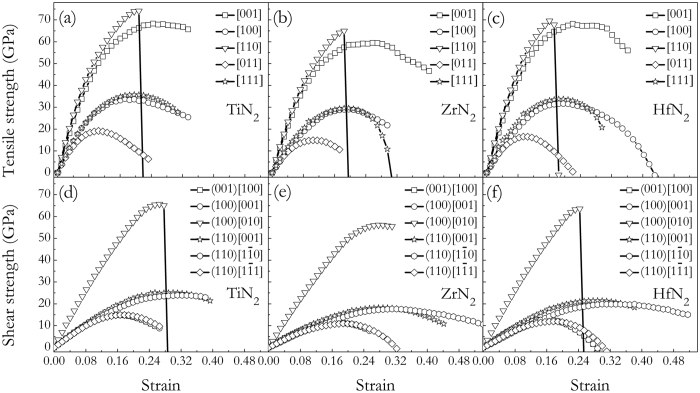
Calculated tensile stress-strain relations for TMN_2_: (**a**) TiN_2_, (**b**) ZrN_2_, (**c**) and HfN_2_. Calculated shear stress-strain relations for TMN_2_: (**d**) TiN_2_, (**e**) ZrN_2_, and (**f**) HfN_2_.

To determine the electronic and structural stabilities as well as the ideal strengths of three TMN_2_ compounds, the stress-strain relations upon tension and shear for tetragonal TMN_2_ phase are calculated in some main crystallographic directions through projection of a 12-atom unit cell onto the corresponding crystal axes with one axis parallel to the strain direction for tension deformation, or with one axis parallel to the slip direction and another axis perpendicular to the slip plane for shear deformation. The schematic of tensile/shear deformation and the ideal strengths deduced from the stress-strain curves for three TMN_2_ compounds are shown in Fig. 7. From Fig. 7(a–c), one can see that the calculated tensile strengths show a similar anisotropy for all three compounds. It shows that all three TMN_2_ have strong stress responses in the [110] directions (TiN_2_: 74.14 GPa, ZrN_2_: 64.95 GPa, HfN_2_: 69.59 GPa) that accord well with their largest directional Young’s moduli (see Fig. 6), which measure the resistance against uniaxial tensions. However, the weakest tensile strength along [011] with the peak tensile stresses below 20 GPa for TMN_2_ (TiN_2_: 19.15 GPa, ZrN_2_: 14.88 GPa, HfN_2_: 16.53 GPa) is much lower than those of 40 GPa for PtN_2_^19^ and 31.1 GPa for TiN^40^ along the [100] directions. The anisotropy ratio of tensile strength (*σ*_max_:*σ*_min_) for TiN_2_ (3.87) is smaller than those of ZrN_2_ (4.36) and HfN_2_ (4.21). Meanwhile, the shear strengths upon large strains for three TMN_2_ are presented in Fig. 7(d–f) in order to further examine the shear deformation where plastic deformation proceeds irreversibly on the atomic scale. First, the highest shear strength for TMN_2_ is found under the (100)[010] direction compare well with their largest shear modulus orientation in the (100) principal shear plane shown in Fig. 6(d–f). Second, the values of the ideal shear strength *τ* and shear strain *γ* of the weakest system is TiN_2_: (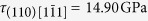, *γ* = 0.166), ZrN_2_: (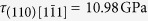, *γ* = 0.188), and HfN_2_: (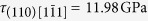, *γ* = 0.177), which is basically lower than that^41^ of TiN: (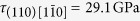, *γ* = 0.21), ZrN: (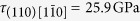, *γ* = 0.17), and HfN: (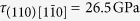, *γ* = 0.15), respectively, showing their lower shear resistance or hardness than these known hard wear-resistant materials. Third, the lowest shear strength of TMN_2_ is lower than the lowest tensile strength. This means the failure mode in tetragonal TMN_2_ phase is dominated by the shear type.

**Figure 8:**
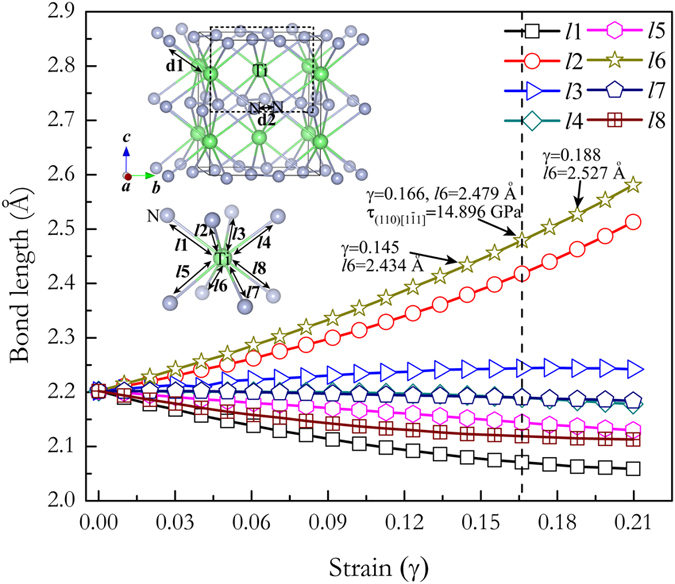
Calculated bond lengths of Ti-N (*ln*) as a function of strain along (110)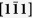 shear directions. The dashed line represents the shear induced structural deformation firstly occurrence.

**Figure 9:**
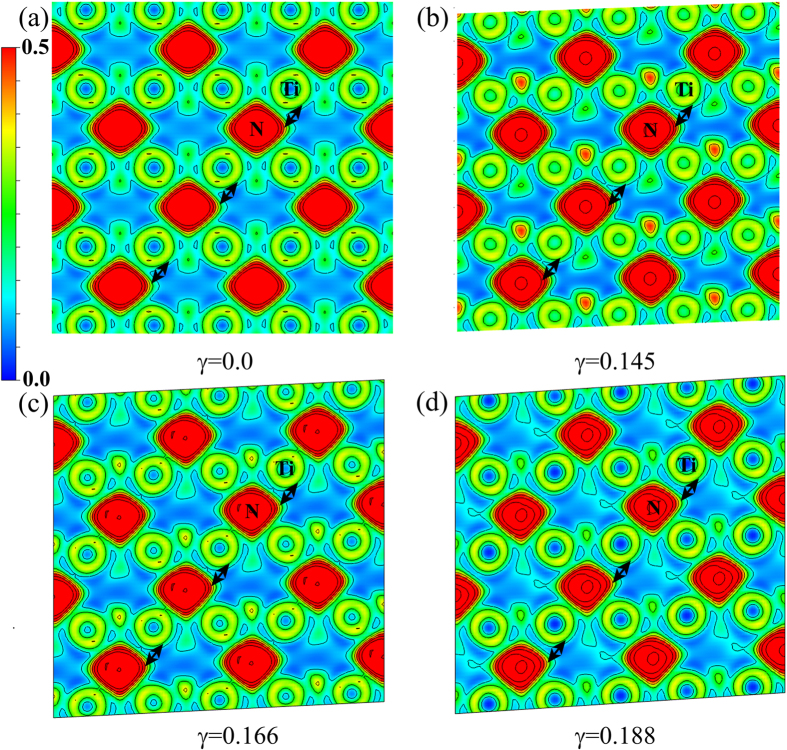
The development of ELF distributions between N-Ti (*l*6 bond) on (110) plane in tetragonal TiN_2_ at selected shear strains (**a**) *γ* = 0.0, (**b**) *γ* = 0.145, (**c**) *γ* = 0.166, and (**d**) *γ* = 0.188.

To further illustrate the atomistic deformation mechanism and the origin of the intriguing bond-breaking pattern of such novel materials in engineering applications, take TiN_2_ for example, we further investigate the variations of bond lengths and electronic structures as a function of applied strain along (110)
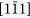
directions. As presented in Fig. 8 where there are two types bond lengths [the Ti-N (2.201 Å) and N-N (1.385 Å) bond length is denoted as *d*1 and *d*2, respectively] in TiN_2_ at equilibrium state. Under increasing shear strains, the N-N lengths denoted as *d*2 remain nearly invariant (*d*2 = 1.385 Å at *γ* = 0 and *d*2 = 1.380 Å at the critical shear strain of *γ* = 0.166). The Ti-N length indicated as *d*1 in TiN_8_ building block is split from one bond distance to eight different bond distances denoted as *ln* (*n* = 1, 2, …) (see the inset in Fig. 8). In Fig. 8, the Ti-N bond lengths indicated as *l*1, *l*4, *l*5, *l*7, and *l*8 decrease in the whole studied shear strain range, on the contrary, the *l*2, *l*3, and *l*6 bonds in TiN_8_ polyhedrons increase conformably at each strain. Especially, the stretched Ti-N bonds denoted as *l*6 increases sharply and breaks at the critical shear strain of *γ* = 0.166, which limits the achievable strengths of TiN_2_. Such a bond-breaking can also be clearly seen from the selected Electronic Localization Function (ELF)^42^,^43^ distributions of TiN_2_ on (110) plane before and after shear instability. At equilibrium state (*γ* = 0, see Fig. 9(a)), a certain electron localization can be seen in the region between adjacent N and Ti atoms indicative of ionic bonding, whereas the electron localization located between N-Ti (*l*6 bonds) atoms decreases gradually upon the incremental shear strains [(110)
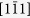
direction] from Fig. 9(c,d). For ELF at strain of *γ* = 0.188 presented in Fig. 9(d), where no electron localized at *l*6 bonds and results in the breaking of this bond. Therefore, the shear-induced structural deformation for tetragonal TiN_2_ can be attributed to the collapse of TiN_8_ polyhedrons by simultaneously breaking of *l*6 bonds, and this is also the case for other two family members of ZrN_2_ and HfN_2_.

## Conclusions

To conclude, the structural, electronic, and mechanical properties as well as the ideal strengths of the recent synthesized tetragonal TiN_2_ and two family members, yet-to-be-synthesized ZrN_2_ and HfN_2_ have been systematically studied by using first-principles calculations. Phonon dispersion and formation enthalpies calculations suggest that three tetragonal TMN_2_ are all dynamically stable at ambient condition and can be synthesizable at readily attainable pressures. The high-incompressible of TMN_2_ is associated with the strong N-N covalent bonding in N_2_ dumbbells and polar covalent bonding between TM and N atoms in TMN_8_ building blocks. However, as compared with known *fcc* TMN, all these tetragonal TMN_2_ exhibit a much larger elastic anisotropy and substantially lower shear strength, which may impose certain limitations on their possible applications. Detailed analyses of the deformed atomic structures under shear strain reveal that the lattice instability of TMN_2_ is due to the collapse of TMN_8_ polyhedrons by simultaneously breaking of TM-N bonds which limits their achievable strength.

## Methods

All first-principles plane wave calculations were performed using the VASP code^44^ in the framework of density functional theory with the generalized-gradient approximation (GGA) proposed by Perdew-Burke-Ernzerhof (PBE) exchange-correlation functional^45^,^46^. The electron-ion interaction was described by the frozen-core all-electron projector augmented wave (PAW) method^47^, which called for a *d*-electron as valence states. The integration in the Brillouin zone for all transition metals dinitrides was employed using the Monkhorst-Pack scheme^48^ (8 × 8 × 6), an energy cutoff of 600 eV for the plane-wave expansions, and a tetrahedron method with Blöch corrections for energy calculations and Gaussian smearing for the stress calculations. The conjugate gradient method was used for the relaxation of structural parameters. Phonon frequencies were calculated using direct supercell^49^, which uses the forces obtained by the Hellmann-Feynaman theorem. Chemical bonding analyses were performed by means of the crystal orbital Hamilton population (COHP) method as implemented in the LOBSTER code^50^,^51^. The independent elastic constants were determined from evaluation of stress tensor generated small strain and bulk modulus, shear modulus, Young’s modulus, and Poisson’s ratio were thus estimated by the Voigt-Reuss-Hill approximation. The stress-strain relationships were calculated by incrementally deforming the model cell in the direction of the applied strain, and simultaneously relaxing the cell basis vectors conjugated to the applied strain, as well as the positions of atoms inside the cell, at each step.

## Additional Information

**How to cite this article**: Zhang, M. *et al*. Electronic bonding analyses and mechanical strengths of incompressible tetragonal transition metal dinitrides TMN_2_ (TM = Ti, Zr, and Hf). *Sci. Rep*. **6**, 36911; doi: 10.1038/srep36911 (2016).

**Publisher's note:** Springer Nature remains neutral with regard to jurisdictional claims in published maps and institutional affiliations.

## Supplementary Material

Supplementary Information

## Figures and Tables

**Table 1:** Calculated Crystal lattices (Å), Unit cell volume (Å^3^), Bond length (Å, *d*_*TM-N*_: *d*_1_; *d*_*N-N*_: *d*_2_), EOS fitted Bulk modulus *B*_0_ (GPa) and its pressure derivative *B*_0_′ for each tetragonal TMN_2_.

TMN_2_	Source	*a*_0_	*c*_0_	*V*_*0*_	*d*_1_	*d*_2_	*B*_0_	*B*_0_'	Δ*H*_*f*_
TiN_2_	This work	4.348	5.321	100.537	2.201	1.385	276	4.362	0.398
	Exp.^15^	4.334	5.294	99.44			385	1.45	
	Theory^15^	4.322	5.269	98.44		1.383	293	3.7	0.386
ZrN_2_	This work	4.596	5.767	121.793	2.354	1.434	257	4.195	0.372
HfN_2_	This work	4.555	5.683	117.854	2.320	1.461	280	4.136	0.328

Also shown is the formation enthalpy (Δ*H*_*f*_, eV/atom).

**Table 2:** Calculated Elastic constants *C*_*ij*_, Bulk modulus *B*, Shear modulus *G*, and Young’s modulus *E* (in units of GPa) for each tetragonal TMN_2_.

Compounds	Source	*C*_11_	*C*_33_	*C*_44_	*C*_66_	*C*_12_	*C*_13_	*B*	*G*	*E*	*v*	*G*/*B*
TiN_2_	This work	520	651	145	343	281	59	276	195	473	0.215	0.707
	Theory^16^	535	653	148	336	279	71	284	197	481	0.218	0.693
ZrN_2_	This work	420	598	111	298	266	70	250	152	379	0.247	0.608
HfN_2_	This work	460	686	126	353	301	68	275	172	423	0.241	0.626
TiN	This work	590		162		123		278	188	460	0.225	0.676
	Exp.^32^	625		163		165						
	Exp.^33^							288				
	Theory^34^	575		163		130		278	185	454		0.665
ZrN	This work	514		121		118		250	147	367	0.253	0.588
	Exp.^35^	471		138		88		215	160		0.16	
	Theory^34^	523		111		116		252	148	371		0.587
HfN	This work	571		114		125		273	150	379	0.269	0.549
	Exp.^35^	679		150		119		306	202		0.15	
	Theory^34^	588		120		113		271	158	397		0.583

Also shown are Poisson’s ratio *v* and *G*/*B* ratio.
