# Supplementary material for: Electronic bonding analyses and mechanical strengths of incompressible tetragonal transition metal dinitrides TMN2 (TM = Ti, Zr, and Hf)
Source: Sci Rep. 2016 Nov 10;6:36911. doi: 10.1038/srep36911 (PMC5103277; doi:10.1038/srep36911)
Supplement: Supplementary Information [file srep36911-s1.pdf]

Supplementary information

Electronic bonding analyses and mechanical strengths of incompressible tetragonal transition metal dinitrides TMN_2 (TM= Ti, Zr, and Hf)

Meiguang Zhang[1], Ke Cheng[2], Haiyan Yan[3], Qun Wei[4] & Baobing Zheng[1]

[1] *College of Physics and Optoelectronic Technology, Nonlinear Research Institute, Baoji University of Arts and Sciences, Baoji 721016, China*, [2] *College of Optoelectronic Technology, Chengdu University of Information Technology, Chengdu 610225, China*, [3] *College of Chemistry and Chemical Engineering, Baoji University of Arts and Sciences, Baoji 721013, China*, [4] *School of Physics and Optoelectronic Engineering, Xidian University, Xi'an 710071, China.*

Correspondence and requests for materials should be addressed to M. G. Z. (zhmgbj@126.com) and Q. W. (weiaqun@163.com)

For the tetragonal crystal, the orientation dependences of the Young's modulus E and shear modulus G which can be determined from the elastic compliance constants s_{ij}, defined as

$$s_{11} = s_{22} = \frac{1}{2}(\frac{C_{33}}{C'} + \frac{1}{C_{11} - C_{12}}) \quad (1)$$

$$s_{12} = \frac{1}{2}(\frac{C_{33}}{C'} - \frac{1}{C_{11} - C_{12}}) \quad (2)$$

$$s_{13} = s_{13} = -\frac{C_{13}}{C'} \quad (3)$$

$$s_{33} = \frac{C_{11} + C_{12}}{C'} \quad (4)$$

$$s_{44} = s_{55} = \frac{1}{C_{44}} \quad (5)$$

$$s_{66} = \frac{1}{C_{66}} \quad (6)$$

where $C' = C_{33}(C_{11} + C_{12}) - 2C_{13}^2$. Based on the calculated s_{ij}, for the tetragonal TMN_2, the Young's modulus E can be expressed as:

$$E^{-1} = s_{11}(\alpha^4 + \beta^4) + s_{33}\gamma^4 + 2s_{12}\alpha^2\beta^2 + 2s_{13}(\beta^2\gamma^2 + \alpha^2\gamma^2)$$
$$+ s_{44}(\beta^2\gamma^2 + \alpha^2\gamma^2) + s_{66}\alpha^2\beta^2 \quad (7)$$

where α, β, and γ is the direction cosine of [*uvw*] direction. The shear modulus G on the (*hkl*) shear plane with shear stress applied along [*uvw*] direction is given by:

$$G^{-1} = 4s_{11}(\alpha_1^2\alpha_2^2 + \beta_1^2\beta_2^2) + 4s_{33}\gamma_1^2\gamma_2^2 + 8s_{12}\alpha_1\alpha_2\beta_1\beta_2 + s_{66}(\alpha_1\beta_2 + \alpha_2\beta_1)^2$$
$$+ 8s_{13}(\beta_1\beta_2\gamma_1\gamma_2 + \alpha_1\alpha_2\gamma_1\gamma_2) + s_{44}[(\beta_1\gamma_2 +_1 \beta_2\gamma_1)^2 + (\alpha_1\gamma_2 + \alpha_2\gamma_1)^2] \quad (8)$$

where α_1, β_1, γ_1, α_2, β_2, γ_2 are the direction cosines of the [*uvw*] and [*HKL*] directions in the coordinate systems, where the [*HKL*] denotes the vector normal to the (*hkl*) shear plane.
